# Social cognitive communication mechanism of digital participation in Chinese traditional music in the context of cultural identity and intangible cultural heritage

**DOI:** 10.3389/fpsyg.2026.1896221

**Published:** 2026-10-07

**Authors:** Tingting Kuai, Jing Qian, Wei Xu

**Affiliations:** 1 College of Culture and Tourism, Jiangsu Vocational Institute of Commerce, Nanjing, Jiangsu, China; 2 School of Cultural Industry and Tourism Management, Sanjiang University, Nanjing, Jiangsu, China; 3 College of Digital Media and Art Design, Jiangsu Vocational Institute of Commerce, Nanjing, Jiangsu, China

**Keywords:** Chinese traditional music, communication mechanism, cultural identity, digital participation, intangible cultural heritage, social cognitive theory

## Abstract

Digital platforms have reshaped how traditional Chinese music circulates, yet the psychological mechanism linking user participation to cultural identification remains undertheorized. Drawing together social cognitive theory, contemporary cultural psychology, and communication theory, this study develops a four-layer architecture (input, participation, cognitive, and identity) coupled with a staged process model spanning information encoding, symbolic decoding, cognitive reconstruction, and identity internalization. A hybrid specification formalizes the mechanism, pairing structural equation modeling with attention-weighted neural aggregation; both components are estimated and validated on the same data. Empirical testing draws on 2,346 behavioral records from music, short-form video, and social media platforms, paired with 1,128 survey responses from users engaged with the guqin, Kunqu, pipa, and regional opera repertoires. Item loadings, heterotrait–monotrait ratios, collinearity diagnostics, and a common-method check document measurement quality. Results indicate that digital participation (DP) shapes cultural identity (CI) mainly through social cognitive communication (SCC), with the co-creation and dissemination strata showing indirect effects that are more than three times those of passive exposure. As the direct participation-identity path remains significant, the pattern is one of partial rather than full mediation. Cultural identity also looks more like a hinge than a terminal state: a chained specification linking identification to downstream cognitive engagement returns a reliable indirect path, which is consistent with the recursive reading the framework proposes but, on single-wave evidence, cannot establish it. Community climate exerts the strongest moderating influence, exceeding both platform affordance and content polish. Since the design is cross-sectional, all of these patterns are read as associations rather than as causal effects; even so, they suggest that audience cultivation and co-creation tooling deserve at least as much investment as interface refinement in the digitization of traditional music.

## 1 Introduction

The accelerated diffusion of digital infrastructure has reshaped how intangible cultural heritage (ICH) circulates, archives itself, and reaches new audiences, opening pathways once confined to physical apprenticeship and regional festivals (Stefano and Davis, 2017). Short-video platforms, immersive media, and algorithmic recommendation now allow heritage forms to surface in everyday feeds. Still, the same mechanisms compress nuance, fragment narrative continuity, and expose fragile traditions to decontextualization (Geismar, 2017).

Within this transformed landscape, Chinese traditional music—encompassing folk song, instrumental traditions, opera, and ritual repertoires—occupies a position of unusual fragility and cultural weight, since its meaning depends as much on embodied performance and communal memory as on sonic content (Zhu, 2024). Recent surveys indicate that although online exposure to guqin, Kunqu, and regional ballads has grown markedly, sustained engagement and intergenerational transmission remain uneven, with younger audiences often treating these forms as aesthetic curiosities rather than living practice (Liu, 2026). That gap between exposure and commitment is, at bottom, a psychological problem: what changes inside a listener between a first encounter and a durable sense of belonging, and under what conditions does the change hold (Yang et al., 2022)?

Scholarly attention to these tensions has expanded along several converging lines. A study on cultural identity (CI)—drawing from the constructivist tradition—treats identification as an ongoing negotiation between symbolic resources and lived experience rather than a static inheritance (Pulis, 2014). Research on digital safeguarding of ICH has examined documentation standards, virtual museums, and participatory archiving, often emphasizing technical feasibility while leaving the audience side under-theorized (Giaccardi, 2012). Communication scholars have meanwhile probed how networked publics form around heritage content, identifying loops of imitation, remix, and affective resonance that differ sharply from broadcast-era reception (Papacharissi, 2015). A parallel literature on traditional music transmission has documented the role of master–apprentice ties, regional ecosystems, and educational policy in sustaining repertoire (Rice, 1987), while a smaller body of study has begun to apply social cognitive theory—particularly observational learning, self-efficacy, and outcome expectancy—to media-mediated cultural behavior (Bandura, 2001a).

Three more recent developments sharpen these questions. Studies of informal digital learning show that online self-efficacy and perceived social presence jointly regulate how much cognitive effort learners invest (Wu, 2023; Pan, 2022). A study on feedback perception indicates that the interpretive frame a learner brings to a platform signals matters at least as much as the signals themselves (Cao and Han, 2024). Contemporary cultural psychology has re-established that cognition, emotion, and motivation are culturally patterned processes rather than neutral substrates onto which culture is later layered (Kitayama and Salvador, 2024).

What these strands have not yet produced is an integrated account of how digital participation (DP) actually shapes cognition and identity in the heritage context. Existing studies tend to isolate a single facet of the problem—platform affordances, audience attitudes, or transmission outcomes—without modeling the recursive process that links them (Wu, 2024). Three questions consequently remain open. Do participatory acts of different cognitive depth carry cultural meaning at different rates, or is participation a single undifferentiated quantity? Does identification, once formed, feed back into the cognitive processing that produced it? And is the translation from participation to identification gated chiefly by technical affordance or by the social climate of the community in which it occurs? Empirical evidence on the mediating role of participatory behaviors—commenting, sharing, remixing, learning—remains sparse and methodologically scattered (Bonacchi and Krzyzanska, 2019), leaving heritage practitioners and platform designers to improvise, with few evidence-based principles to guide content strategy, interaction design, or community cultivation.

These three questions set the agenda for what follows, and they are psychological before they are technological. With digital media now woven into daily routines of listening, learning, and self-presentation, the mechanism at issue concerns observational learning, efficacy beliefs, motivational uptake, and the developmental study of identity formation—processes that unfold inside individuals even when their traces are recorded as platform metrics (Branje, 2022). Building on social cognitive theory, contemporary cultural psychology, and communication theory, the study develops a multidimensional model linking platform affordances, user participation behaviors, cognitive and affective processing, and cultural identification, then tests that model against survey and behavioral data drawn from heritage-oriented digital communities (Cao, 2023).

Three contributions follow, each stated as a claim that the data can refute rather than as a synthesis of the literature. The first is that participation is stratified in its cultural consequences: acts differing in cognitive investment—watching, reacting, remaking, redistributing—should transmit to identity at sharply different rates, which converts a familiar descriptive typology into a testable ordering. The second is that identity may be re-entrant rather than terminal. Once identification takes hold, it should begin to organize the very cognitive processing that produced it, a proposition an additive framework cannot express because they close the system at outcome measurement. Whether that loop actually runs in time is a question the present design can pose but not settle; what a single wave of data can show is whether the association such a loop implies is present at all. The third is that the translation is socially rather than technically gated, so community climate should outweigh interface affordance in shaping the participation-to-identity path—a prediction that runs counter to the technocentric emphasis of much digital safeguarding work.

Taken together, these claims extend social cognitive theory into a domain where the modeled behavior is cultural rather than instrumental, and they return to cultural psychology a measurable account of how mediated interaction reshapes belonging. The remainder of the article sets out the theoretical foundations, model construction, empirical strategy, results, and their wider implications.

## 2 Theoretical foundations and research framework

### 2.1 Cultural identity and the communication of intangible cultural heritage

Cultural identity, as a theoretical construct, has migrated across disciplines over the past half-century, moving from essentialist accounts that treated it as a stable inheritance to relational and processual formulations that read it as continually rewritten through symbolic interaction (O'Neill and Townsend, 2002). Hall (sHall1996) articulation of identity as both “being” and “becoming” remains a productive anchor, since it allows researchers to hold together the sedimented layers of tradition and the fluid moments of self-positioning that characterize contemporary cultural life. For heritage scholarship, this dual character matters: ICH is at once an inheritance to be safeguarded and a resource to be enacted, and the boundary between preservation and reinvention is rarely as clean as policy documents imply (Smith, 2006).

Contemporary cultural psychology adds a constraint that heritage scholarship has been slow to absorb: culture and mind are mutually constituted, so cultural materials do not merely furnish content for cognition but organize the perceptual, emotional, and motivational processes through which content is registered at all (Kitayama and Salvador, 2024). On that reading, an encounter with heritage music is never a neutral intake of sound; it is patterned by schemas the culture has already installed.

Within the ICH context, identity construction unfolds along several intersecting dimensions—cognitive recognition of heritage symbols, affective attachment to communal memory, behavioral participation in practice, and reflexive narration of belonging. Chinese traditional music carries these layers with unusual density. A guqin piece is not only a sonic object; it indexes literati aesthetics, regional lineage, and ritualized listening habits that have accumulated over centuries (Yung, 1997). When such repertoire circulates, listeners encounter compressed cultural memory whose interpretation depends on prior exposure, social cues, and the symbolic frames offered by mediating institutions (Sterne, 2009). Prior cultural exposure, therefore, functions as an interpretive resource rather than as a demographic control, since it determines which features of a performance become perceptually available in the first place. Heritage-experience research reports this pattern precisely, with prior involvement conditioning the depth of the mental experience through which identification is assembled (Yang et al., 2022).

To formalize this layered relationship, the cultural identity strength of an individual *i* toward a heritage form can be expressed as a weighted aggregation of dimensional components; a standard composite formulation in identity research is expressed as follows:


$$\begin{array}{l} CI_{i}=\overset{n}{\underset{k=1}{\sum }}w_{k}\cdot D_{ik} \end{array}$$ (1)

where *D*_*ik*_ denotes the score of individual *i* on identity dimension *k* (cognitive, affective, behavioral, and narrative), and *w*_*k*_ the dimension weight derived from empirical loading.

The link between identity and heritage communication is itself dynamic. Each communicative episode revises the symbolic stock available for future identification, producing what is best described as a recursive loop, and digital environments plausibly accelerate it. Adapting the classic diffusion logic to this recursive case, the temporal evolution of aggregate identification within a digitally mediated audience may be written as follows (Equation 2):


$$\begin{array}{l} \frac{dCI\left(t\right)}{dt}=\alpha \cdot E\left(t\right)\cdot \left[CI_{\max}-CI\left(t\right)\right]-\beta \cdot CI\left(t\right) \end{array}$$ (2)

where *E*(*t*) captures the intensity of digital exposure and participation, α the assimilation rate, β the decay rate under attentional competition, and *CI*_max_ the saturation ceiling, following standard adoption-decay dynamics. The formulation makes explicit why platform-driven engagement, rather than mere content availability, has become the pivotal variable in how traditional music sustains identification in everyday digital life (Van Dijck et al., 2018). It should be read as an analytic statement of the dynamics the mechanism implies, not as a model fitted here: a cross-sectional design identifies only the stationary counterpart of such a system, a restriction to which Section 3.3 returns.

### 2.2 Social cognitive theory and the mechanism of digital participation

Bandura's social cognitive theory advances a triadic reciprocal model in which personal factors, behavior, and environmental conditions co-determine one another, departing from earlier stimulus–response accounts by granting human agency a constitutive role (Bandura, 2001b). Translated into digital settings, the “environment” is no longer a neutral backdrop but an algorithmically curated feed whose recommendations, visibility cues, and interaction affordances continuously reshape what users notice, attempt, and internalize (Bucher, 2018). Four mechanisms inherited from the original theory acquire new texture under such conditions: observational learning thrives on the abundance of demonstration videos and live streams; symbolic modeling intensifies through avatar-mediated reenactment; visible feedback metrics recalibrate self-efficacy beliefs; and outcome expectancies are formed not only through anticipated personal benefit but through perceived social currency within networked communities (LaRose, 2010).

Recent evidence from digital learning environments supports the reading with some precision: online self-efficacy and perceived social presence jointly predict the effort learners invest, and the influence of platform feedback depends on how learners construe it rather than on how often it arrives (Wu, 2023; Cao and Han, 2024; Pan, 2022). Heritage participation differs in content but not in structure, which is what licenses the transfer of the theory to this setting.

The triadic interaction can be written in dynamic form, following Bandura's standard formulation, as follows (Equation 3):


$$\begin{array}{l} P\left(t+1\right)=f\;\left[P\left(t\right),B\left(t\right),E\left(t\right)\right] \end{array}$$ (3)

where *P*, *B*, and *E* denote personal cognition, behavior, and environment at time *t*, and *f* a coupling function capturing reciprocal influence.

Digital participation is best treated as a layered construct rather than a flat behavioral variable. Four ascending strata can be distinguished—exposure (*L*_1_), interaction (*L*_2_), co-creation (*L*_3_), and dissemination (*L*_4_)—each demanding different cognitive investment and yielding different feedback signals (van Dijck, 2009). A composite participation index *DP*_*i*_ for user *i* aggregates these strata with empirically derived weights as expressed in the following (Equation 4):


$$\begin{array}{l} DP_{i}=\overset{4}{\underset{j=1}{\sum }}\lambda_{j}\cdot L_{ij},\;\overset{4}{\underset{j=1}{\sum }}\lambda_{j}=1 \end{array}$$ (4)

where *L*_*ij*_ is the standardized score of user *i* at participation stratum *j* and λ_*j*_ the weight reflecting that stratum's cognitive demand, derived through confirmatory factor loading in the manner standard for formative composite measurement (Hair et al., 2018).

Coupling these layers to social cognitive formation requires a mediating mechanism rather than a direct path. The specification adopted here holds that digital participation acts on social cognition, *SC*, through self-efficacy, *SE*, and outcome expectancy, *OE*, with environmental affordance *A* moderating the gain, as expressed in the following (Equation 5):


$$\begin{array}{l} SC_{i}=\beta_{0}+\beta_{1}\cdot SE_{i}\left(DP_{i}\right)+\beta_{2}\cdot OE_{i}\left(DP_{i}\right)+\beta_{3}\cdot A_{i}\cdot DP_{i}+\varepsilon_{i} \end{array}$$ (5)

Following the moderated-mediation specification widely adopted in social cognitive research (Hayes, 2022). The expression makes explicit that exposure alone is insufficient; cognitive uptake depends on whether participatory acts generate efficacy gains and credible expectancy signals within an enabling platform environment (Schunk and Usher, 2019). Motivationally, this is the difference between a platform that shows users what others have made and one that lets them discover what they can make themselves; only the second generates the mastery information that informs revisions to efficacy beliefs (Cao and Han, 2024). This framework supplies the analytical scaffolding for the model construction that follows.

### 2.3 Research framework and variable definition

Drawing the two preceding strands together, this study specifies a research framework organized around three core constructs: digital participation (DP), social cognitive communication (SCC), and cultural identity (CI). Digital participation is treated as the antecedent driver, social cognitive communication as the mediating mechanism, and cultural identity as the distal outcome, with platform affordance entering as a contextual moderator (Bonacchi et al., 2019). The framework rests on the logic that participatory acts in heritage-oriented digital communities generate cognitive and affective traces that, in turn, rework the symbolic resources from which identification is assembled (Banta, 2018). What distinguishes the specification from earlier participation-identity models is not the presence of a mediator, which is by now conventional, but the treatment of participation as ordered by cognitive depth and of identity as potentially re-entrant. Both restrictions are stated sharply enough to fail, though only the first can be decisively tested on data gathered at a single point in time.

Operationally, *DP* retains the four-stratum specification introduced earlier (exposure, interaction, co-creation, and dissemination). *SCC* is decomposed into three observable facets: cognitive elaboration (*CE*), affective resonance (*AR*), and normative endorsement (*NE*), each measured through validated multi-item scales adapted to the heritage music setting (Angelillo, 2025). *CI* is captured through cognitive recognition, affective attachment, behavioral commitment, and narrative articulation, consistent with the composite expression in Equation 1. Item wordings were adapted from published instruments and re-anchored to the heritage music setting; the full item pool, together with the translation and back-translation record, is described in Section 4.1.

The mediating role of *SCC* between *DP* and *CI* is formalized as follows:


$$\begin{array}{l} SCC_{i}=\gamma_{0}+\gamma_{1}\cdot DP_{i}+\underset{m}{\sum }\delta_{m}X_{im}+\epsilon_{i} \end{array}$$ (6)

$$\begin{array}{l} CI_{i}=\theta_{0}+\theta_{1}\cdot DP_{i}+\theta_{2}\cdot SCC_{i}+\underset{m}{\sum }\phi_{m}X_{im}+\nu_{i} \end{array}$$ (7)

where *X*_*im*_ denotes control covariates (age, prior musical training, and platform tenure), and ϵ_*i*_, ν_*i*_ are stochastic disturbances. The mediating effect is the product γ_1_θ_2_, following Baron and Kenny's classical decomposition adapted for structural equation modeling (SEM) estimation (Baron and Kenny, 1986).

The following is an expression of a moderated path that captures the conditioning role of affordance *A*:


$$\begin{array}{l} CI_{i}=\theta_{0}+\theta_{1}DP_{i}+\theta_{2}SCC_{i}+\theta_{3}(SCC_{i}\times A_{i})+ \end{array}\begin{array}{l} \underset{m}{\sum }\phi_{m}X_{im}+\nu_{i} \end{array}$$ (8)

The interaction term *SCC*_*i*_×*A*_*i*_ tests whether platforms offering richer participatory affordances amplify the translation of cognitive engagement into identification, following standard moderation specification (Aiken and West, 1991).

From this structure, four hypotheses follow. H1: *DP* positively predicts *CI*. H2: *SCC* mediates the *DP*−*CI* relation. H3: Among the four participation strata, co-creation (*L*_3_) and dissemination (*L*_4_) exert stronger indirect effects on *CI* than exposure (*L*_1_). H4: Platform affordance *A* moderates the *SCC*−*CI* link, with stronger effects under high-affordance conditions. These hypotheses are tested in the empirical analysis reported in Section 4.

## 3 Construction of the social cognitive communication mechanism model for digital participation in Chinese traditional music

### 3.1 Overall architecture design of the digital participation communication mechanism

The architecture developed here joins social cognitive theory's triadic logic with the processual reading of cultural identity, organizing the mechanism into four hierarchically nested layers—input, participation, cognitive, and identity—threaded by a moderating affordance band and a closing feedback loop (Bao and Yu, 2018). Each layer carries a distinct functional load while remaining recursively coupled to its neighbors, so that communication of Chinese traditional music in digital settings is treated as a stratified flow rather than a linear transmission (Poell et al., 2019).

The input layer aggregates the symbolic and technical antecedents that seed the mechanism: heritage repertoire (guqin pieces, opera arias, and regional ballads), digitization standards, creator outputs, and the recommender logic that governs visibility. These inputs do not act directly on users; they pass through participation affordances that decide which sonic objects become candidates for engagement (Helberger et al., 2018). The participation layer then channels users into the four ascending strata introduced earlier—exposure, interaction, co-creation, and dissemination—producing a behavioral substrate captured by the composite index *DP*_*i*_ in Equation 4.

What gives the architecture its analytical bite is the cognitive layer above participation. Behavioral traces are translated into cognitive elaboration, affective resonance, and normative endorsement, with self-efficacy and outcome expectancy serving as internal calibrators that filter which experiences gain symbolic weight (Schunk and DiBenedetto, 2021). The identity layer, finally, accumulates these cognitive deposits into the four-dimensional identification profile specified in Equation 1, feeding back into participation through revised motivation and selective attention. Within the architecture, this loop is constitutive rather than decorative: identification is modeled as a continuously updated disposition that biases subsequent participatory choices (Macdonald, 2013). The status of that claim warrants careful consideration. It belongs, for the moment, to the model rather than to the evidence, since cross-sectional data can reveal the association such a loop implies but cannot observe the loop unfolding. Read psychologically, the proposal is identity formation in the developmental sense—exploration through participation, commitment through repeated confirmation, reconsideration when the community's signals change (Branje, 2022).

Figure 1 maps the architecture and its principal pathways.

**Figure 1:**
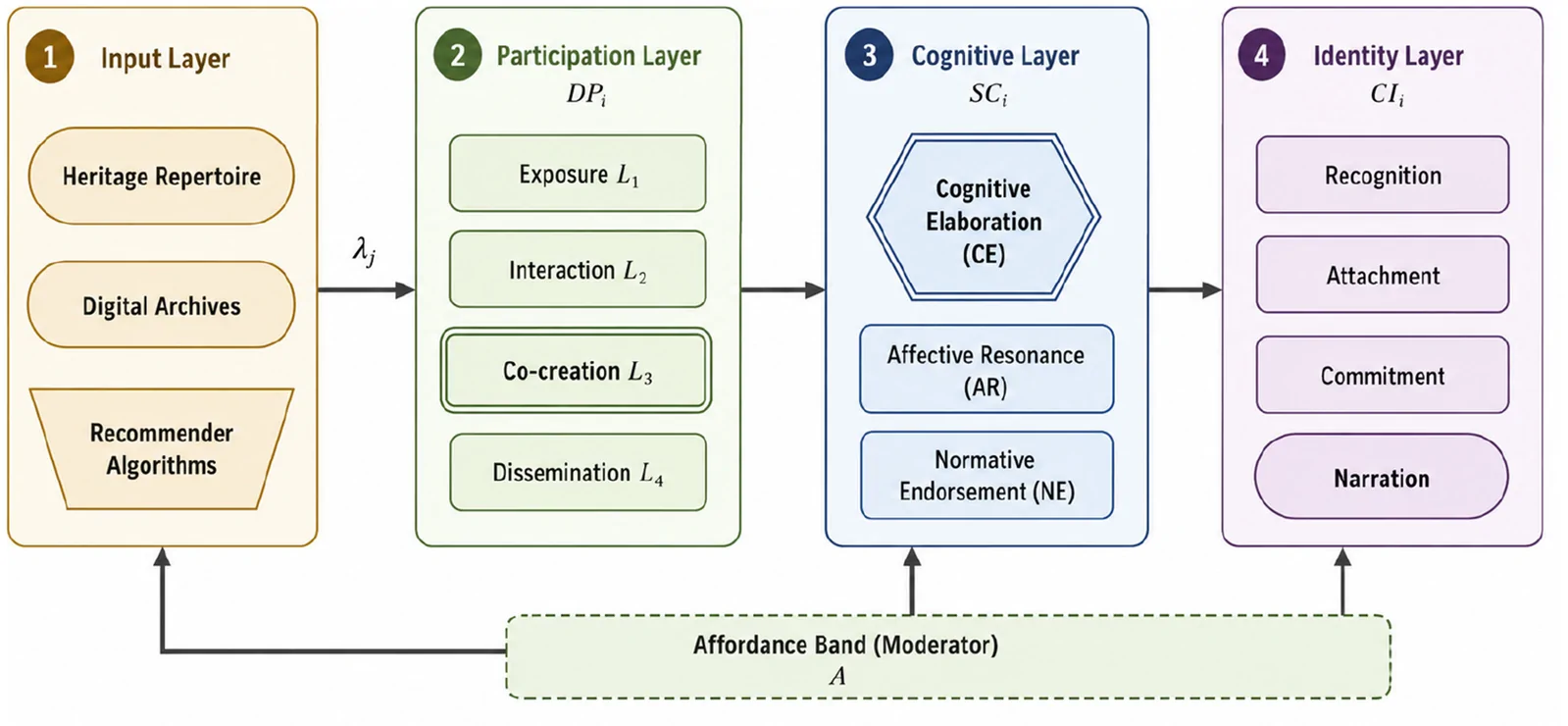
Principle diagram of the four-layer social cognitive communication mechanism, showing forward translation pathways (input → participation → cognition → identity), the affordance band moderating cross-layer gain, and the recursive feedback loop that re-seeds the input layer. The feedback arrow states a theoretical proposition of the architecture; it is not temporally identified by the cross-sectional data analyzed here. DP, digital participation; SCC, social cognitive communication; CI, cultural identity; CE, cognitive elaboration; AR, affective resonance; NE, normative endorsement.

Table 1 presents the layered structure, specifying constituent elements, functional roles, and key variables that anchor the subsequent measurement.

**Table 1:** Hierarchical structure and functional positioning of the digital participation communication mechanism.

Layer	Core elements	Functional role	Key variables
Input	Heritage repertoire, digital archives, creator outputs, and recommender algorithms	Supply symbolic and technical antecedents	Content richness and algorithmic visibility
Participation	Exposure, interaction, co-creation, and dissemination	Convert exposure into behavioral substrate	*L*_1_-*L*_4_ scores and *DP*_*i*_ index
Cognitive	Cognitive elaboration, affective resonance, and normative endorsement	Translate behavior into symbolic uptake	*CE*, *AR*, *NE*; *SE*, and *OE*
Identity	Recognition, attachment, commitment, and narration	Aggregate cognition into identification	*CI*_*i*_ composite and weights *w*_*k*_
Affordance band	Interface design, community norms, and visibility cues	Moderate cross-layer translation gain	Affordance index *A*
Feedback loop	Revised motivation and algorithmic re-ranking	Re-seed input with updated signals	Loop gain and decay rate β

Two design choices distinguish the architecture from earlier accounts. Treating affordance as a cross-cutting moderator rather than a layer-specific attribute lets the model accommodate platform heterogeneity without inflating its parametric burden. Reading the feedback loop as a constitutive element, rather than closing the system at the level of outcome measurement, captures the cumulative drift of repeated digital encounters that descriptive heritage studies have noted but rarely formalized (Dottorini et al., 2026). Figure 1 should therefore be read as an estimation map rather than as an illustration. Its forward arrows correspond one-to-one with parameters recovered below: the participation-to-cognition translation is the first-stage coefficient of Equation 6, the cognition-to-identity translation the second-stage coefficient of Equation 7, the affordance band the interaction term of Equation 8, and the feedback loop the chained specification examined—though not temporally identified—in Section 4.3. This stratified scaffolding underwrites the variable specification and structural estimation that follow in Sections 3.2 and 3.3.

### 3.2 Multidimensional digital participation–social cognition coupling communication process

Having fixed the layered architecture in Section 3.1, the argument now turns to the process side: how a user actually moves over time from a first encounter with a digitized piece of traditional music to a stabilized act of cultural identification. The flow unfolds across four coupled stages—information encoding, symbolic decoding, cognitive reconstruction, and identity internalization—each shaped by an interplay of platform, content form, community interaction, and algorithmic recommendation (Guo, 2023).

Encoding begins when creators, archivists, or heritage institutions translate sonic and performative material into platform-native formats: short videos, livestream fragments, immersive audio, interactive scores. Format choice is far from neutral; a 15-s Douyin clip and a 30-min Bilibili lecture encode the same guqin piece into radically different cognitive openings (Cantor, 2019). Decoding follows as the recommender surfaces the encoded artifact to a user whose interpretive resources—prior exposure, musical training, peer cues—determine whether the symbolic payload registers as ornament, curiosity, or invitation (Schäfer, 2011). Community interaction at this point matters more than is usually granted: a thread of informed comments can reframe an opaque passage into a culturally legible one within seconds.

Cognitive reconstruction sits at the pivot of the process. Decoded fragments are stitched into the user's existing schemas through elaboration, affective tagging, and normative comparison with what peers are seen to value (Bolin, 2017). The dynamics of this stitching can be written, following standard reinforcement formulations, as follows (Equation 9):


$$\begin{array}{l} C_{i}\left(t+1\right)=C_{i}\left(t\right)+\eta \cdot \left[R_{i}\left(t\right)-C_{i}\left(t\right)\right] \end{array}$$ (9)

where *C*_*i*_(*t*) is the cognitive state of the user *i* at time *t*, *R*_*i*_(*t*) the reconstruction signal generated by the current participatory episode, and η a learning rate sensitive to affordance richness and emotional salience (Sutton and Barto, 2018). Internalization, the final stage, occurs when reconstructed cognition consolidates into a disposition stable enough to bias future selection, sharing, and self-narration—closing the loop back to Section 3.1's feedback channel (Gee, 2013).

Figure 2 traces the staged flow and its principal couplings.

**Figure 2:**
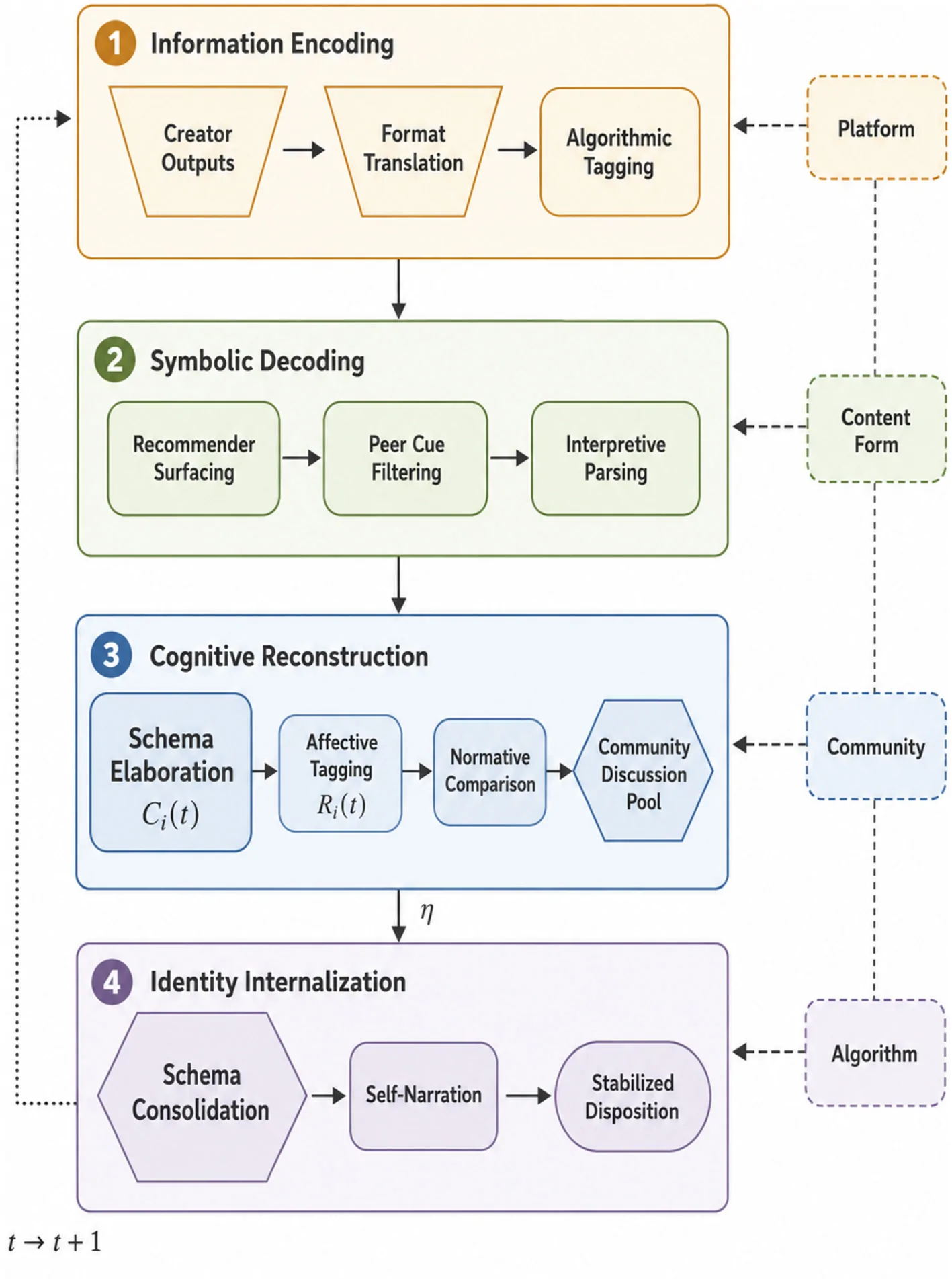
Stage flow of the digital participation–social cognition coupling process, showing encoding, decoding, reconstruction, and internalization, with platform, content form, community, and recommender inputs acting laterally on each transition. The four stages are analytic positions within the coupling process rather than measured time points. *C*_*i*_, cognitive state of user; *R*_*i*_, reconstruction signal; η, learning rate.

Table 2 presents the distinguishing features of each stage to their dominant cognitive outputs.

**Table 2:** Stage characteristics of digital participation and corresponding cognitive outputs.

Stage	Dominant driver	User behavior	Cognitive output	Identity trace
Information encoding	Creator, archive, and format design	Passive reception of pushed content	Attention capture and schema priming	Latent recognition cue
Symbolic decoding	Recommender + peer cues	Browsing and light reaction (like and save)	Interpretive parsing and salience tagging	Awareness fragment
Cognitive reconstruction	Community interaction	Commenting, learning, and remixing	Elaboration, affective resonance	Emerging attachment
Identity internalization	Self-reflection and narrative practice	Sharing, teaching, and repertoire selection	Normative endorsement and schema consolidation	Stabilized identification
Recursive feedback	Updated motivation	Selective re-engagement	Reinforced cognitive bias	Identification accrual

Two features of the process deserve emphasis. The transitions are asymmetric: encoding-to-decoding is high-throughput but low-conversion, whereas reconstruction-to-internalization is low-throughput but high-conversion, which means platform strategies aimed solely at exposure volume tend to leave the most consequential transition underserved (Gee, 2013). The lateral inputs—platform, content form, community, and algorithm—do not act uniformly across stages; community weight rises sharply at reconstruction, while algorithmic gating dominates the earlier transitions. Figure 2 is likewise tied to the estimation rather than free-standing. Its four stages are not measured as separate time points; they enter the empirical model as the salience weights recovered by the attention block and reported in Section 4.3, where the pivotal status claimed for reconstruction becomes a quantity that can be checked rather than asserted. This staged, asymmetric, laterally conditioned flow is what the structural model in Section 3.3 will operationalize.

### 3.3 Mathematical modeling and algorithmic implementation of core mechanism elements

The staged process specified in Section 3.2 invites a more compact mathematical treatment, one that can be estimated against survey and behavioral data without losing the layered structure of the mechanism. The specification draws on structural equation modeling for the latent-variable backbone and cognitive diffusion dynamics for the temporal extensions, and concludes with an attention-weighted aggregation suited to heterogeneous participation traces (Kline, 2023). As a hybrid formalism invites the suspicion that some of its parts are decorative, the division of labor is worth stating at the outset: the latent-variable block carries the hypothesis tests, the attention block is estimated and validated against held-out data in Section 4.3, and the continuous-time expressions define the dynamic system of which the estimated cross-sectional model is a snapshot. Which of these are fitted to data, and which remain analytic scaffolding, is made explicit at the close of this section.

The digital participation intensity for user *i* at time *t* is specified as a saturating function of stratum-weighted behavioral inputs as expressed as follows (Equation 10):


$$DP_{i}(t)=\frac{\sum_{j=1}^{4}\lambda_{j}L_{ij}(t)}{1+\kappa \cdot \sum_{j=1}^{4}L_{ij}(t)}$$ (10)

with κ controlling diminishing returns, following standard saturation specification (Bass, 2004). The following logistic-type equation governs the diffusion of social cognition across an interacting user population (Equation 11):


$$\begin{array}{l} \frac{dSC_{i}\left(t\right)}{dt}=\mu \cdot DP_{i}\left(t\right)\cdot \left[SC_{\max}-SC_{i}\left(t\right)\right]-\rho \cdot SC_{i}\left(t\right) \end{array}$$ (11)

where μ is the assimilation coefficient, ρ the attentional decay, and *SC*_max_ the cognitive ceiling. Peer-driven contagion is layered on through a network term as expressed in the following (Equation 12):


$$\begin{array}{l} \Delta SC_{i}\left(t\right)=\xi \underset{k\in N\left(i\right)}{\sum }a_{ik}\cdot \left[SC_{k}\left(t\right)-SC_{i}\left(t\right)\right] \end{array}$$ (13)

with *a*_*ik*_ the tie strength between users *i* and *k*, following standard network-diffusion form (Centola, 2018).

Cultural identity convergence is then captured by an exponential approach to a moving target shaped by sustained cognitive input as expressed in the following (Equation 13):


$$\begin{array}{l} CI_{i}\left(t\right)=CI_{\max}\cdot \left(1-e^{-\tau \overset{t}{\underset{0}{\int }}SC_{i}\left(s\right)\;ds}\right) \end{array}$$ (13)

with τ the convergence rate. The following expression signifies the heterogeneous participatory signals that are fused through a multilayer perceptron (MLP)–style hidden representation:


$$\begin{array}{l} h_{i}=\sigma \;\left(W_{2}\cdot \sigma \left(W_{1}x_{i}+b_{1}\right)+b_{2}\right) \end{array}$$ (14)

where *x*_*i*_ stacks the stratum scores, *W*_1_ *and W*_2_ are weight matrices, *b*_1_ *and b*_2_ are biases, and σ the rectified linear unit (ReLU) activation in the standard MLP formulation (Goodfellow et al., 2016). Attention weighting then re-scores stages by salience as expressed in the following:


$$\begin{array}{l} \alpha_{is}=\frac{\exp(e_{is})}{\sum_{s\prime =1}^{S}\exp(e_{is^{\prime }})},\;e_{is}=v^{⊤}\tanh(W_{a}h_{is}) \end{array}$$ (15)

producing the weighted propagation effect provided as follows:


$$\begin{array}{l} \Pi_{i}=\overset{S}{\underset{s=1}{\sum }}\alpha_{is}\cdot h_{is} \end{array}$$ (16)

which feeds the outcome layer through the following equation:


$$\begin{array}{l} \hat{C}I_{i}=w_{o}^{⊤}\Pi_{i}+b_{o} \end{array}$$ (17)

For the latent-variable backbone, parameter estimation follows maximum likelihood under the SEM measurement specification, which is expressed as follows (Equation 18):


$$\begin{array}{l} \Sigma \left(\theta \right)=\Lambda \Phi \Lambda^{⊤}+\Theta_{\delta } \end{array}$$ (18)

with Λ the loading matrix, Φ the latent covariance, and Θ_δ_ the residual covariance, in classical Jöreskog form (Jöreskog et al., 2016). End-to-end training of the neural pathway minimizes a regularized loss and expressed as follows:


$$\begin{array}{l} ℒ=\frac{1}{N}\sum_{i=1}^{N}{\left(CI_{i}-\hat{C}I_{i}\right)}^{2}+\gamma \cdot {\left(\theta \right)}^{2} \end{array}$$ (19)

optimized through an adaptive stochastic gradient method with early stopping on a held-out fold (Falbel, 2021). Table 3 inventories the core variables and symbols for clarity before estimation.

**Table 3:** Core model variables and symbol specification.

Symbol	Meaning	Type
*DP*_*i*_(*t*)	Digital participation intensity for user *i*	Composite
*L*_*ij*_	Stratum score (exposure–dissemination)	Observed
λ_*j*_	Stratum weight	Parameter
*SC*_*i*_(*t*)	Social cognition state	Latent
μ, ρ	Assimilation, decay coefficients	Parameter
*a*_*ik*_	Peer tie strength	Network input
*CI*_*i*_(*t*)	Cultural identity convergence	Outcome
α_*is*_	Attention weight on stage *s*	Learned
Π_*i*_	Weighted propagation effect	Derived
Θ_δ_	SEM residual covariance	Parameter

This hybrid specification keeps the SEM block answerable to theory while letting the attention-weighted block absorb non-linearities a purely linear path would otherwise push into the residual. Two clarifications guard against overreading it. The components actually fitted to data are the measurement and structural equations, the mediation and moderation decompositions, and the attention-weighted aggregation, whose implementation, training protocol, and out-of-sample performance are reported in Section 4.3. The continuous-time expressions for diffusion, contagion, and convergence are analytic scaffolding: they state the dynamic system the mechanism implies, but a cross-sectional design identifies only its stationary counterpart, so their parameters are not estimated here. The recursive reading of identity belongs to that scaffolding as well. What Section 4.3 tests is the association the loop would produce, not the loop itself, and this restriction is explicitly carried forward into the limitations stated in Section 6.

## 4 Empirical analysis and discussion of results

### 4.1 Data collection and sample characteristics

Empirical material for testing the mechanism was assembled from two complementary streams between March and October 2025. The behavioral stream draws on publicly observable user traces from three categories of platforms—digital music services (NetEase Cloud Music and QQ Music), short-video platforms (Douyin, Kuaishou, and Bilibili), and social media (Weibo and Xiaohongshu). Candidate accounts were identified through platform search and hashtag crawling using a fixed keyword list covering guqin, Kunqu, pipa, erhu, and regional opera repertoires. They were retained only if they had interacted at least three times with content carrying those tags during the observation window. That threshold was set to exclude one-off algorithmic encounters while remaining low enough to keep genuinely passive viewers inside the frame (Guo, 2023). Sampling was purposive rather than probabilistic, so the resulting sample represents engaged heritage-music audiences on Chinese platforms rather than the general population.

The survey stream complements these traces with a structured questionnaire administered to identifiable users who consented to participate, capturing the latent constructs—self-efficacy, outcome expectancy, cognitive and affective processing, and the four identity dimensions—that behavioral logs cannot reach. Invitations were issued via platform direct message to all 2,346 accounts in the behavioral pool, and 1,128 usable responses were returned, an effective response rate of 48.1%. All items were measured on seven-point Likert scales. Participation items were adapted from established measures of user-generated content engagement, efficacy and expectancy items from social cognitive instruments, and identity items from established cultural identification scales, each re-anchored to the heritage music setting. English source items were translated into Chinese and independently back-translated by a bilingual researcher who had not seen the originals; discrepancies were resolved through discussion before piloting on 60 respondents, whose data are excluded from the analytic sample.

After deduplication, bot filtering, and removal of inactive accounts, 2,346 valid behavioral records were retained, of which 1,128 were paired with completed questionnaires through a hashed account identifier. Preprocessing followed a four-step pipeline: log cleaning, semantic tagging of content categories, standardization of stratum-level participation scores, and missing-value imputation using expectation-maximization for items with less than 5% missingness (Little and Rubin, 2019).

Two elements of that pipeline deserve fuller description, since they carry the majority of the interpretive weight. Semantic tagging was performed by two trained coders working independently from a codebook that assigned each observed act to one of the four participation strata and each content item to a repertoire category. The coders overlapped on a random 20% of the corpus. They returned chance-corrected agreement of 0.87 for stratum assignment and 0.84 for repertoire category, with remaining disagreements adjudicated by the third author.

Stratum scores were then standardized within platform before aggregation, so that platform-specific base rates of liking and commenting would not be mistaken for differences in participation depth, and the stratum weights entering the composite index were fixed by confirmatory factor loading rather than assigned a priori. Analyses were conducted in R with lavaan for the structural model and in Python with PyTorch for the attention block.

Both data streams involved human participants, and the ethics arrangements differed between them. The Research Ethics Committee of Jiangsu Vocational Institute of Commerce reviewed and approved the full protocol (Reference Number: JSIC-IRB-2025-0317). Questionnaire respondents provided electronic informed consent on the instrument's opening page, before any item was displayed; the consent text outlined the purpose of the study, the voluntary nature of participation, the right to withdraw at any point without consequence, and the anonymous storage of responses. For the behavioral stream, the committee approved a waiver of individual consent, on the grounds that the traces were publicly posted, non-sensitive, and collected in line with the platforms' terms of service. Account identifiers were hashed at the time of collection; no private messages or direct identifiers were retained; and all results are reported in aggregate, so that no individual user can be identified from the subsequent analyses.

Reliability and validity checks returned satisfactory indicators. Cronbach's α for the participation, cognition, and identity scales ranged from 0.84 to 0.91, and composite reliability ranged from 0.86 to 0.93. Convergent validity, assessed through average variance extracted, exceeded the 0.50 threshold across all latent constructs, while discriminant validity was confirmed under the Fornell–Larcker criterion (Hair et al., 2019).

Table 4 summarizes the sample's demographic spread and stratum-level participation. Two patterns stand out: younger users dominate the active strata, and formal musical training correlates with markedly higher composite participation, suggesting that pre-existing cultural capital still gates depth of engagement even in algorithmically open environments.

**Table 4:** Sample demographic profile and digital participation distribution.

Attribute	Category	*n*	%	Mean *DP*_*i*_
Gender	Female/male	1,302/1,044	55.5/44.5	0.58/0.54
Age	18–25	894	38.1	0.62
Age	26–35	765	32.6	0.59
Age	36–50	487	20.8	0.49
Age	>50	200	8.5	0.41
Training	Formal musical background	612	26.1	0.67
Tenure	>3 years on platform	1,488	63.4	0.61
Stratum	Co-creators (*L*_3_ active)	538	22.9	0.74

As Figure 3 illustrates, demographic composition varies sharply by platform type; Figure 4 makes clear that the strata of participation are unevenly distributed, a heterogeneity that the structural analysis in Section 4.2 must absorb (Amaral et al., 2024).

**Figure 3:**
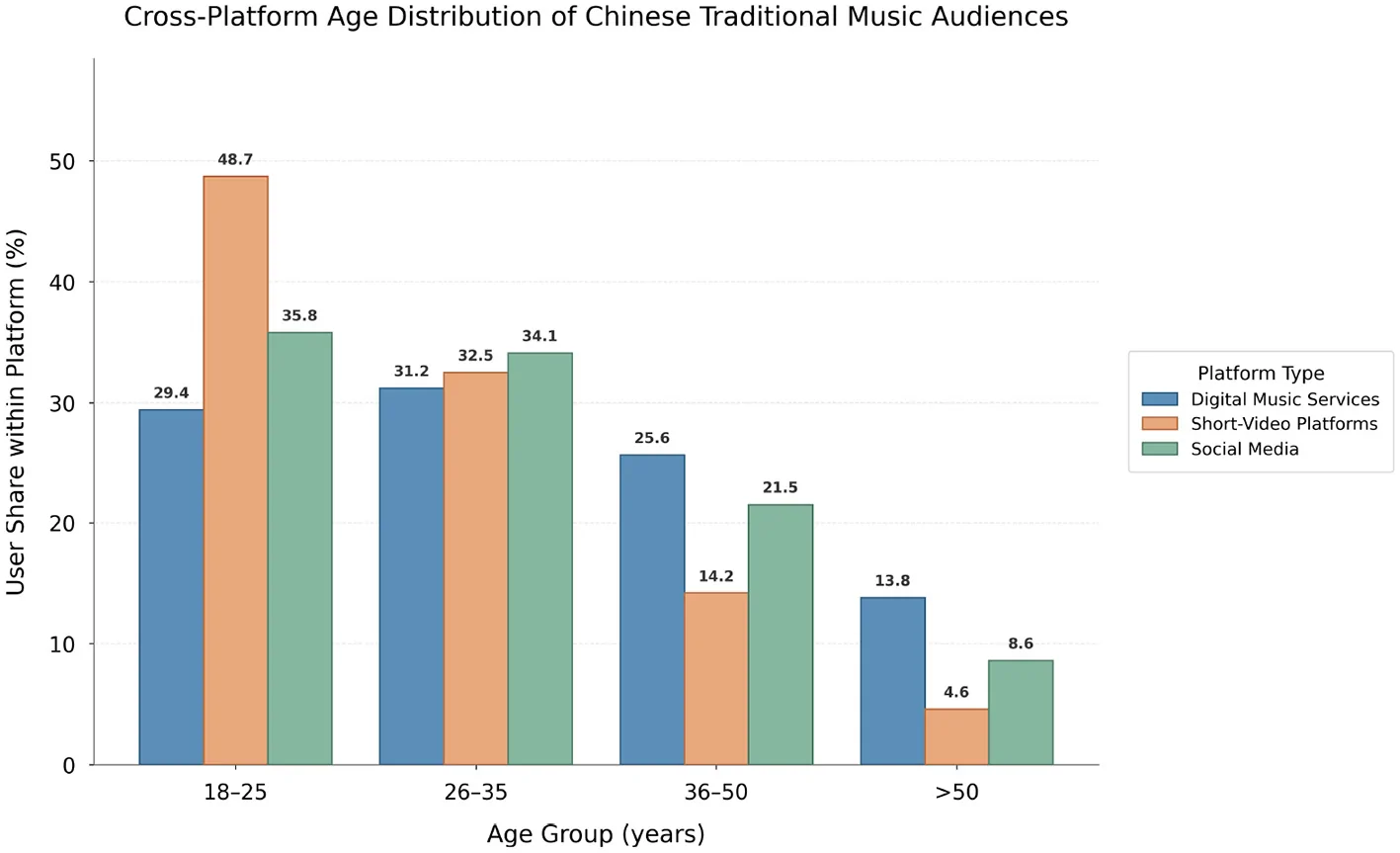
Cross-platform demographic distribution showing age-group shares across the three platform categories, with short-video platforms skewing youngest and digital music services retaining a broader age spread.

**Figure 4:**
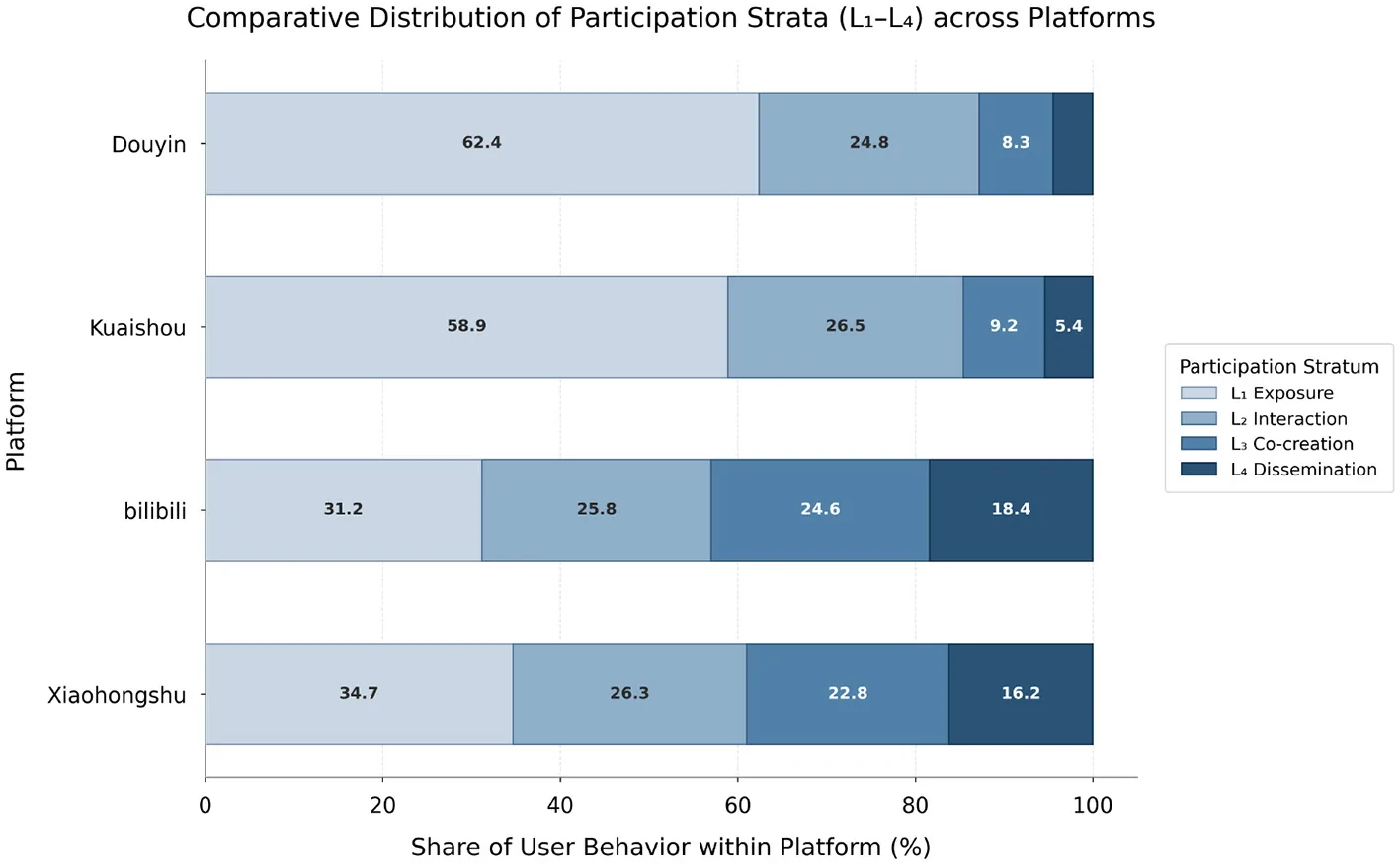
Comparative distribution of stratum-level participation (*L*_1_−*L*_4_) by platform, revealing that co-creation and dissemination cluster on Bilibili and Xiaohongshu, while exposure-only behavior dominates Douyin and Kuaishou.

Because a structural model is only as trustworthy as the measurement model beneath it, the measurement properties are reported in full before any path is interpreted. Table 5 gives, for each latent construct, the number of retained items, the range of standardized loadings, composite reliability, average variance extracted, and the largest variance inflation factor among its indicators. Every loading clears 0.65 and is significant at the 0.001 level; composite reliability ranges from 0.86 to 0.93; average variance extracted exceeds 0.50 throughout; and no variance inflation factor approaches the conventional threshold of 3.3, so multicollinearity does not distort the coefficients reported later. The confirmatory measurement model fits acceptably, with a chi-square-to-degrees-of-freedom ratio of 2.18, Root Mean Square Error of Approximation (RMSEA) of 0.045, Standardized Root Mean Square Residual (SRMR) of 0.041, Comparative Fit Index (CFI) of 0.957, and Tucker–Lewis Index (TLI) of 0.949.

**Table 5:** Measurement model assessment: item loadings, reliability, convergent validity, and collinearity diagnostics.

Construct	Items	Loading range	CR	AVE	Max VIF
Digital participation (DP)	12	0.682–0.841	0.89	0.54	2.31
Cognitive elaboration (CE)	5	0.714–0.862	0.90	0.64	2.07
Affective resonance (AR)	5	0.706–0.849	0.88	0.60	1.94
Normative endorsement (NE)	4	0.691–0.833	0.86	0.61	1.88
Self-efficacy (SE)	4	0.735–0.871	0.91	0.68	2.15
Outcome expectancy (OE)	4	0.702–0.848	0.89	0.63	2.02
Cultural identity (CI)	12	0.674–0.879	0.93	0.58	2.44
Platform affordance (A)	5	0.688–0.827	0.87	0.57	1.76
Content quality	4	0.699–0.836	0.86	0.60	1.69
Community climate	4	0.721–0.858	0.90	0.66	1.83

AVE, average variance extracted; Max VIF, variance inflation factor.

Discriminant validity was assessed twice, since the Fornell–Larcker criterion alone is known to miss violations that the heterotrait–monotrait ratio detects (Henseler et al., 2015; Voorhees et al., 2016). Table 6 reports the heterotrait–monotrait ratios above the diagonal, the square root of average variance extracted on the diagonal, and the inter-construct correlations below it. No ratio exceeds 0.72, comfortably under the 0.85 benchmark, and each diagonal entry exceeds the correlations in its row and column, so the constructs are empirically distinct.

**Table 6:** Discriminant validity: heterotrait–monotrait ratios (above diagonal), square root of average variance extracted (diagonal), and construct correlations (below diagonal).

Construct	DP	CE	AR	NE	CI	A
DP	0.735	0.571	0.524	0.498	0.531	0.437
CE	0.512	0.800	0.672	0.621	0.659	0.451
AR	0.468	0.601	0.775	0.648	0.627	0.418
NE	0.441	0.557	0.583	0.781	0.612	0.403
CI	0.476	0.594	0.561	0.548	0.762	0.412
A	0.389	0.402	0.371	0.358	0.365	0.755

Common method bias was addressed procedurally and statistically. Behavioral and self-report sources were kept separate, item order was randomized, and anonymity was assured at the point of administration. A marker-variable test using a theoretically unrelated construct left the substantive path coefficients essentially unchanged, and the first unrotated factor accounted for 28.4% of the variance, well-below the level at which method variance would be a plausible rival explanation (Podsakoff et al., 2024). With measurement quality established on these several fronts, the structural estimates that follow can be read as relations among constructs rather than as artifacts of the instrument.

### 4.2 Empirical test of digital participation effects on social cognitive communication

Building on the measurement model validated above, the structural model specified in Equations 6–8 was estimated on the paired sample of 1,128 respondents using maximum likelihood with Satorra-Bentler robust standard errors, after confirming acceptable model fit (χ^2^/*df* = 2.31, RMSEA = 0.048, CFI = 0.951, and TLI = 0.942) under the cutoffs recommended for SEM applications in communication research (Hu and Bentler, 1999). Direct and indirect pathways were decomposed using the standard mediation expression provided as follows (Equation 20):


$$\begin{array}{l} Indirect_{DP\to CI}=\gamma_{1}\cdot \theta_{2} \end{array}$$ (20)

with bias-corrected bootstrap confidence intervals based on 5,000 resamples. Multi-group analysis was then run across three contrasts—participation stratum, music type (instrumental, vocal/opera, and ritual), and age cohort—using the chi-square difference test on nested invariance models (Cheung et al., 2024).

The full-sample estimates anchor the picture. The direct effect of *DP* on *SCC* returned γ_1_ = 0.482 (*p* < 0.001); *SCC* on *CI* returned θ_2_ = 0.413 (*p* < 0.001); and the residual direct *DP*→*CI* path stood at θ_1_ = 0.187. As the direct path remains significant, indirect effect of 0.199, alongside a total effect of 0.386, accounts for roughly 52% of the total effect, indicating partial rather than full mediation. In the terminology of Zhao and colleagues, direct and indirect effects share a sign, so the pattern is more precisely described as complementary mediation, and the label is used consistently in this sense throughout Sections 4.2 and 4.3 (Zhao et al., 2010). The moderating interaction *SCC*×*A* produced θ_3_ = 0.156 (*p* = 0.002), supporting H4. Effect sizes were summarized through the following expression (Equation 21):


$$\begin{array}{l} f^{2}=\frac{R_{full}^{2}-R_{reduced}^{2}}{1-R_{full}^{2}} \end{array}$$ (21)

yielding *f*^2^ = 0.21 for the mediator block, a medium effect under Cohen's convention (Lachenbruch, 1989).

Stratum decomposition delivered the more revealing contrast. Indirect effects through *SCC* rose sharply from exposure (*L*_1_: 0.071) and interaction (*L*_2_: 0.142) to co-creation (*L*_3_: 0.246) and dissemination (*L*_4_: 0.231), confirming H3. The pairwise difference between *L*_3_ and *L*_1_ was tested using the following expression (Equation 22):


$$\begin{array}{l} z=\frac{{\hat{\beta }}_{L_{3}}-{\hat{\beta }}_{L_{1}}}{\sqrt{SE_{L_{3}}^{2}+SE_{L_{1}}^{2}}} \end{array}$$ (22)

returning *z* = 4.86 (*p* < 0.001).

The music-type comparison, presented in Table 7, shows that instrumental repertoires (guqin, pipa, and erhu) yield stronger indirect transmission than vocal and opera forms, a gap that plausibly reflects the lower interpretive entry barrier for sonically immersive instrumental clips on short-video feeds. Age-group analysis gave the inverse pattern, with the cohort comparison ratio (Equation 23):


$$\begin{array}{l} \Delta_{age}=\frac{\beta_{18-35}-\beta_{>50}}{\beta_{>50}}=0.38 \end{array}$$ (23)

indicating that younger users channel participation into cognition with substantially greater efficiency, though their baseline identification level remains lower than that of older cohorts.

**Table 7:** Path coefficient estimates and hypothesis test results.

Path	Estimate	SE	*t*-value	Decision
*DP*→*CI* (direct)	0.187	0.041	4.56^***^	H1 supported
*DP*→*SCC*	0.482	0.038	12.68^***^	–
*SCC*→*CI*	0.413	0.045	9.18^***^	–
*DP*→*SCC*→*CI*	0.199	0.024	–	H2 supported
*L*_3_ indirect via *SCC*	0.246	0.029	8.48^***^	H3 supported
*L*_1_ indirect via *SCC*	0.071	0.022	3.23^**^	H3 supported
*SCC*×*A*→*CI*	0.156	0.050	3.12^**^	H4 supported
Instrumental music	0.221	0.034	6.50^***^	–
Vocal/opera music	0.178	0.039	4.56^***^	–

Read together, Figures 5, 6 support the hierarchical logic anticipated in Section 3.3: depth of participation, not breadth, tracks cognitive uptake, and platform affordance appears to amplify the conversion rather than to substitute for it (Gayo, 2025).

**Figure 5:**
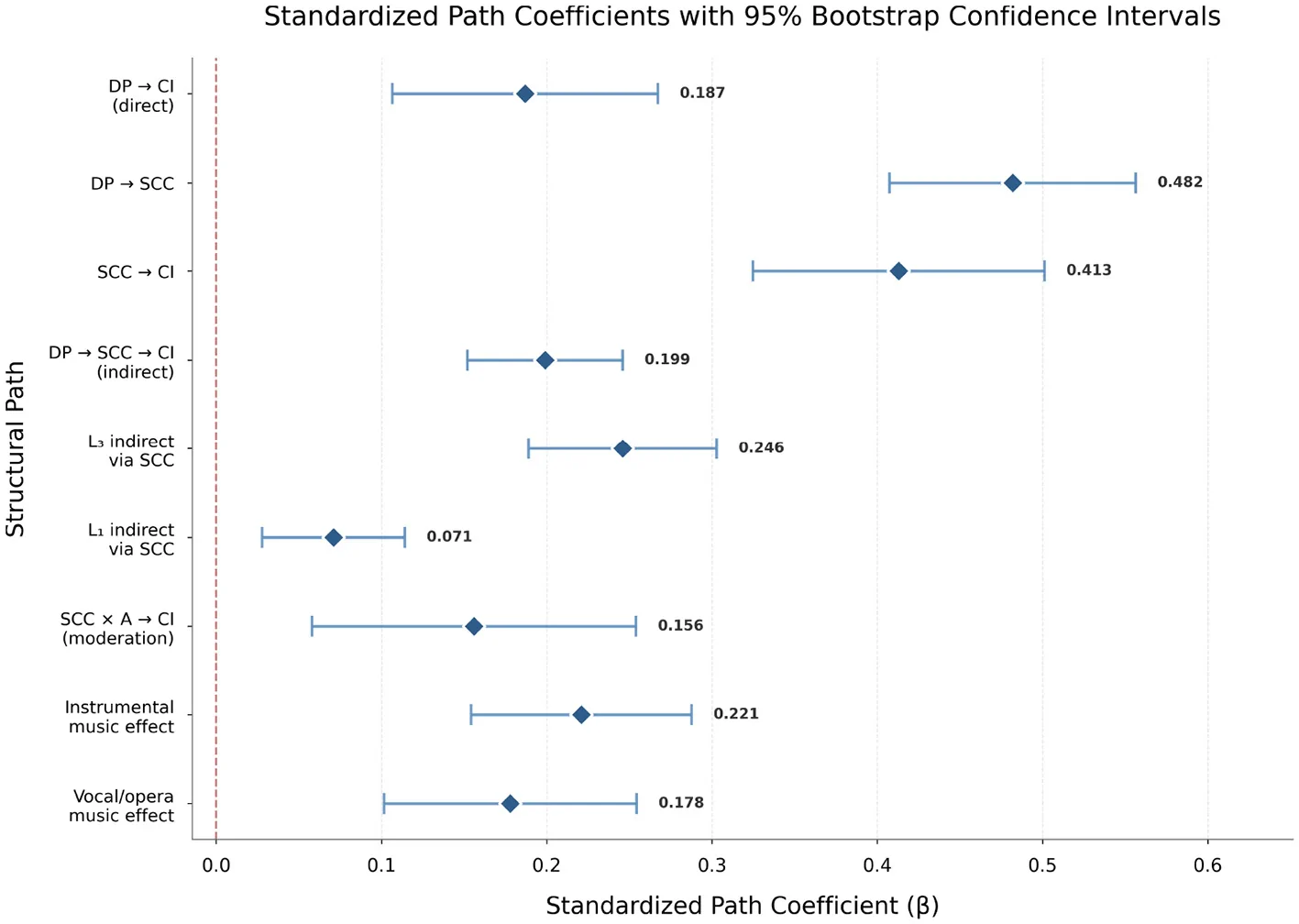
Standardized path coefficients across the structural model, with bootstrap 95% confidence intervals overlaid on each estimate to show pathway robustness.

**Figure 6:**
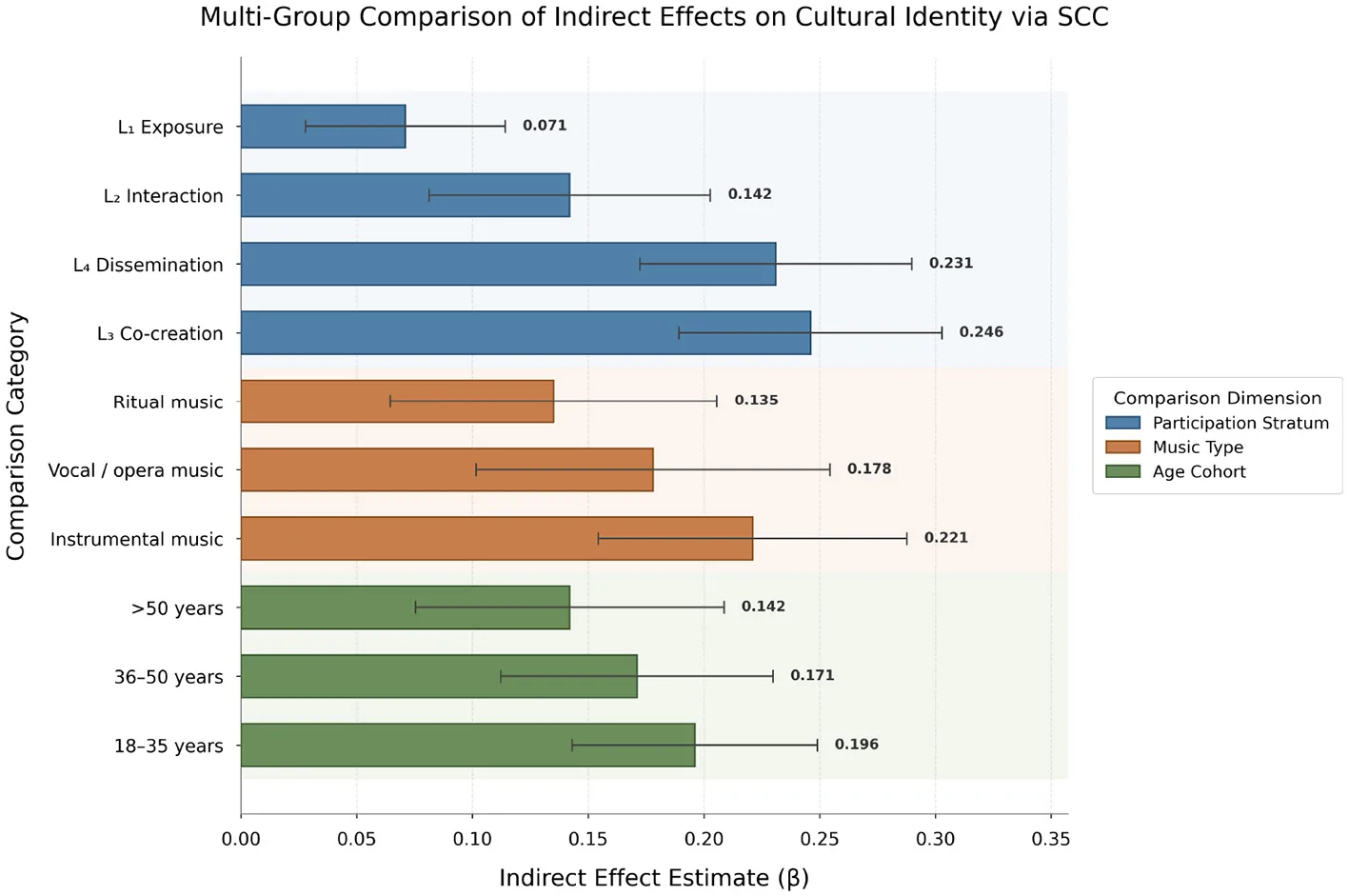
Multi-group comparison of indirect effects across participation strata, music types, and age cohorts, with *L*_3_/*L*_4_ strata and instrumental music registering the strongest cognitive transmission.

### 4.3 Mediation and moderation analysis of the cultural identity construction mechanism

The mediation logic posited in Section 2.3 is here subjected to a more demanding test. The model was re-specified with cultural identity treated alternately as an endogenous outcome and as an internal mediator nested within the *SCC*→*CI* pathway, then examined how platform type, content quality, and community climate condition the transmission. Bootstrap mediation followed the standard product-of-coefficients approach with 5,000 resamples (Preacher and Hayes, 2008), and conditional indirect effects were derived through (Equation 24):


$$\begin{array}{l} CIE\left(M\right)=\left(\gamma_{1}+\gamma_{3}M\right)\cdot \left(\theta_{2}+\theta_{4}M\right) \end{array}$$ (24)

with *M* denoting the moderator and γ_3_, θ_4_ the corresponding interaction coefficients (Hayes and Rockwood, 2020). The Johnson–Neyman technique was applied to locate moderator regions of significance (Equation 25):


$$\begin{array}{l} M^{*}=\frac{-\left(\gamma_{1}\theta_{4}+\gamma_{3}\theta_{2}\right)\pm \sqrt{\Delta }}{2\gamma_{3}\theta_{4}} \end{array}$$ (25)

where Δ is the discriminant of the conditional effect equation, following Hayes' formulation (Hayes and Montoya, 2017).

The mediation block returned a stable indirect effect of *DP* on *CI* through *SCC* at 0.199 (95% CI [0.151, 0.247]). Since the direct path retains significance, this is again partial, complementary mediation rather than the full mediation that a non-significant direct effect would indicate (Zhao et al., 2010). A second specification then treated cultural identity itself as an internal mediator between cognitive elaboration and behavioral commitment; the chained indirect path yielded an estimate of 0.124 (95% CI [0.089, 0.163]). That estimate is consistent with the re-entrant role sketched in Section 2.3, yet it is worth being blunt about what it cannot do. All variables in the chain were measured at a single point in time, so the ordering comes from the model rather than from the data, and cross-sectional estimates of mediated effects are known to diverge substantially from their longitudinal counterparts (Maxwell and Cole, 2007). The same coefficient would arise if users who already identify strongly simply elaborate more, which is why the result is reported here as an association compatible with recursion rather than as evidence of it.

Moderation results sharpen the picture. Platform type interacted with *SCC* at θ_4_ = 0.183 (*p* < 0.001), with high-affordance platforms (Bilibili and Xiaohongshu) yielding conditional indirect effects roughly 1.7 times those of low-affordance feeds. Content quality, indexed by production polish and informational depth, moderated the first-stage path at γ_3_ = 0.142 (*p* = 0.004). Community climate, measured through reciprocity and supportive tone, produced the largest second-stage gain at θ_4_ = 0.211 (*p* < 0.001), with the effect size expressed as Equation 26:


$$\begin{array}{l} \Delta R_{mod}^{2}=R_{interaction}^{2}-R_{main}^{2} \end{array}$$ (26)

returning $\Delta R_{mod}^{2}=0.087$ across the three moderators jointly (Aguinis et al., 2017). Robustness was checked against alternative model specifications, with consistency index:


$$\begin{array}{l} \rho_{robust}=\frac{1}{K}\overset{K}{\underset{k=1}{\sum }}\frac{\left|{\hat{\beta }}_{k}-\bar{\beta }\right|}{\bar{\beta }}\leq 0.10 \end{array}$$ (27)

across *K* = 6 subsamples, indicating stable parameter recovery.

Table 8 makes the conditioning pattern legible: community climate registers the largest moderating weight, exceedingly even platform affordance—a ranking that runs counter to the technocentric expectation and that the remainder of the analysis therefore probes rather than assumes.

**Table 8:** Mediation and moderation test results comparison.

Path/effect	Estimate	95% CI	*p*	Decision
*DP*→*SCC*→*CI* (partial, complementary mediation)	0.199	[0.151, 0.247]	< 0.001	Supported
*DP*→*CE*→*CI*→*BC* (chained)	0.124	[0.089, 0.163]	< 0.001	Supported
Platform type moderation	0.183	[0.108, 0.258]	< 0.001	Supported
Content quality moderation (1st-stage)	0.142	[0.045, 0.239]	0.004	Supported
Community climate moderation (2nd-stage)	0.211	[0.137, 0.285]	< 0.001	Supported
High-affordance conditional indirect	0.276	[0.214, 0.338]	< 0.001	Stronger
Low-affordance conditional indirect	0.162	[0.108, 0.216]	< 0.001	Weaker
High-quality content × *DP*	0.318	[0.241, 0.395]	< 0.001	Stronger
Warm community climate × *SCC*	0.347	[0.268, 0.426]	< 0.001	Strongest
Robustness consistency ρ_*robust*_	0.078	–	–	Stable

Two notes keep the table honest. Its first row reports partial, complementary mediation, not full mediation: the direct *DP*→*CI* coefficient in Table 7 remains significant at 0.187, the total effect is 0.386, and the indirect effect accounts for roughly 52% of it. Its final row reports the mean relative deviation of parameter estimates across six subsamples, so the value of 0.078 falls below the 0.10 tolerance set in Equation 27 rather than above the fit threshold.

Figures 7, 8 together carry the theoretical payoff. The mechanism is robust, but its conversion efficiency is socially rather than technically gated: where communities cultivate reciprocity, and content carries interpretive depth, the participation-to-identification translation accelerates, regardless of platform polish.

**Figure 7:**
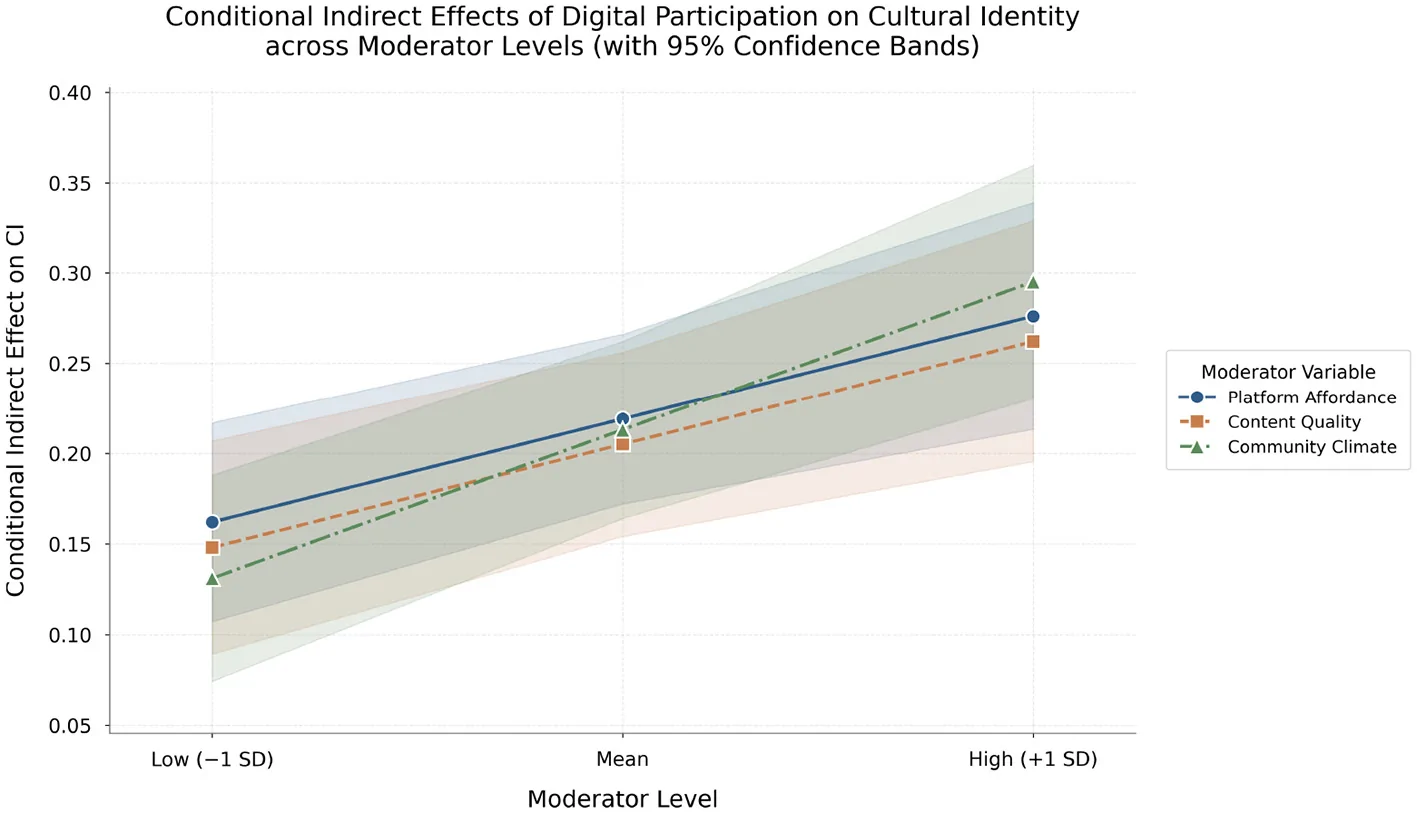
Conditional indirect effects of digital participation on cultural identity across low, mean, and high levels of each moderator, with overlapping confidence bands showing where moderation shifts cross significance thresholds.

**Figure 8:**
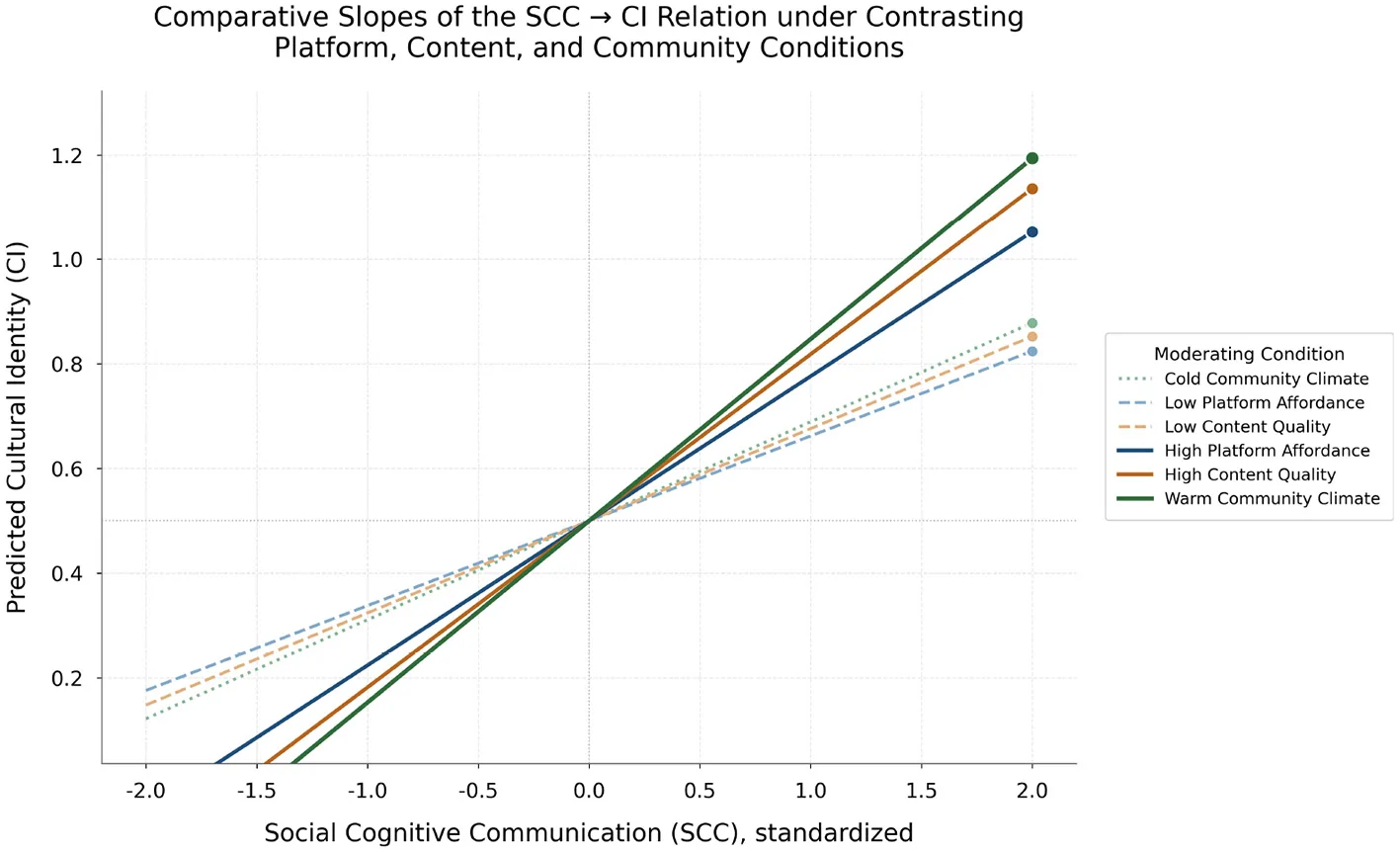
Comparative slopes of the *SCC*→*CI* relation under contrasting platform, content, and community conditions, revealing the steepest gains in warm-community settings rather than on the most technically rich platforms.

A hybrid model earns its second component only by demonstrating that the component adds something, so the attention-weighted block specified in Equations 14–17 was implemented and validated rather than left as a formal gesture. Stratum scores, platform indicators, and stage-level behavioral aggregates were stacked as inputs to a two-layer perceptron with 16 hidden units and rectified linear activation; the attention layer scored the four process stages, and the weighted representation was mapped to the identity outcome through a linear read-out. Training minimized the regularized objective of Equation 19 under Adam, with the paired sample split into five folds, an inner validation split for early stopping, and regularization strength chosen by grid search on that inner split alone, so no test fold informed model selection. Figure 9 reports the comparison of interest.

**Figure 9:**
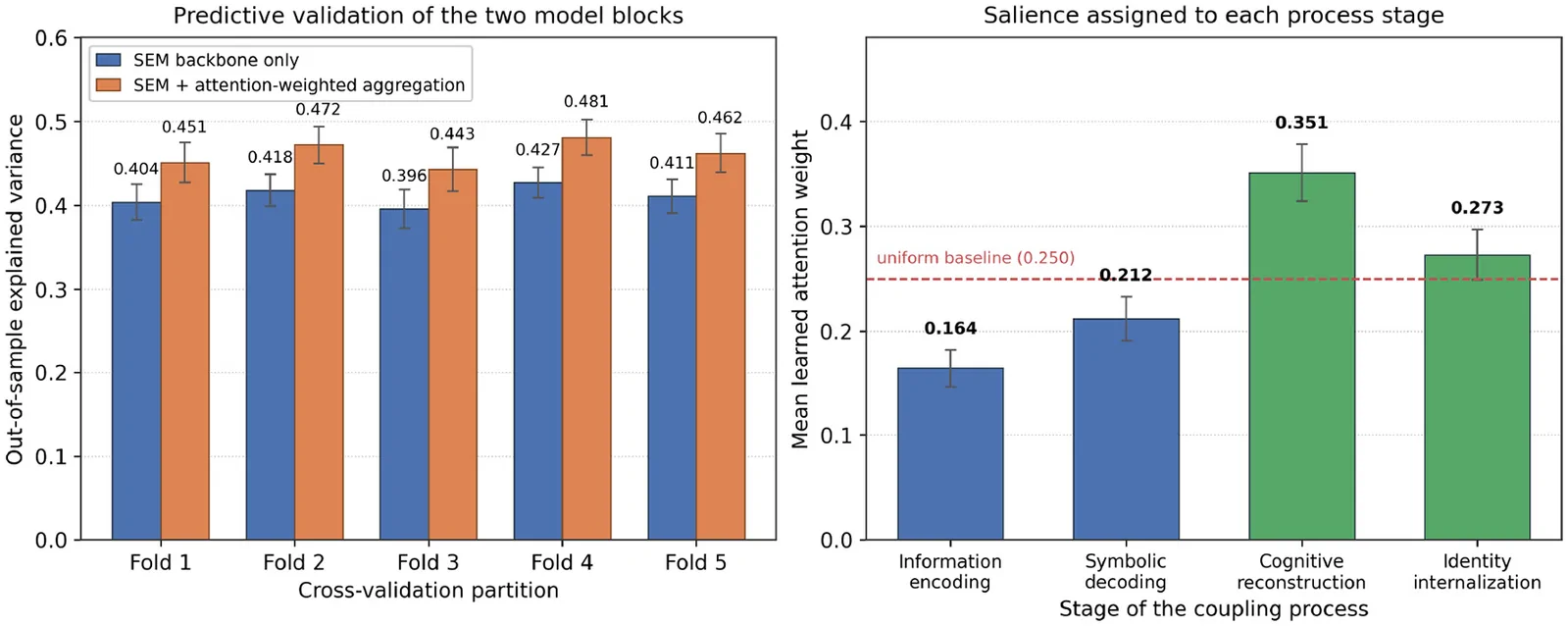
Validation of the attention-weighted block against the structural backbone, showing out-of-sample explained variance across five cross-validation partitions for each specification and the mean attention weight assigned to each stage of the coupling process. The consistent predictive gain indicates that the neural component contributes information rather than redundancy. At the same time, the salience of cognitive reconstruction independently corroborates the pivotal transition identified in the structural results.

Two things follow. Out-of-sample explained variance rises from a mean of 0.411 for the structural model alone to 0.462 for the augmented specification, a modest but consistent gain of roughly five percentage points that holds across all folds; the attention block therefore captures non-linearity the linear path leaves in the residuals without displacing the theoretical model. The learned weights, moreover, are far from uniform: cognitive reconstruction absorbs 0.351 of the total saliences against 0.164 for information encoding. That the two blocks, estimated under different assumptions, agree on where the mechanism concentrates is the strongest internal check the design affords.

## 5 Discussion

The empirical pattern that emerged across Sections 4.2 and 4.3 supports a reading that goes beyond confirming the hypothesized paths. Three interlocking observations organize what follows; the findings are then set against prior study and against the alternative explanations this design cannot rule out.

First, digital participation appears to drive social cognitive transmission not through the volume of exposure but through the depth to which observational learning, symbolic modeling, and community feedback interweave. The sharp rise in the indirect effect from the exposure stratum to co-creation, from 0.071 to 0.246, suggests that watching a Kunqu clip and remixing one are categorically different acts. The first leaves a transient perceptual trace; the second engages symbolic modeling in Bandura's strict sense, since the user must select, reorganize, and reproduce elements of the heritage form, generating mastery information and outcome feedback that exposure alone never supplies.

This is where the psychological reading earns its keep: enactive experience of remaking a phrase is precisely the source from which efficacy beliefs are revised, and the digital-learning literature documents the same asymmetry between passive reception and productive effort (Wu, 2023; Cao and Han, 2024). Community interaction during co-creation then supplies the corrective and reinforcing cues that turn imitation into internalized practice—a result sitting awkwardly with the still-common assumption that platform reach is itself a proxy for cultural transmission.

Second, cultural identity looks more like a hinge than an endpoint. The chained mediation estimate of 0.124 is consistent with a reading in which identification not only accrues from cognitive engagement but also comes to organize it, filtering which guqin pieces a user pauses on, which comments they trust, which lineages they begin to claim. Consistency, however, is not sequence. A single wave of data cannot separate that reading from its mirror image, in which prior identification simply prompts deeper elaboration, and the methodological literature warns that cross-sectional estimates of such chains can be badly misleading about the longitudinal process they are taken to represent (Maxwell and Cole, 2007). Read through contemporary cultural psychology, the proposal is that culture and mind mutually constitute one another on short time scales: cultural material supplies schemas, schemas organize attention, and reorganized attention selects further cultural material (Kitayama and Salvador, 2024). It remains, on this evidence, a proposal.

It also implies that identity here takes a context-bound form—among younger Douyin users, an aestheticized self-presentation; among Bilibili learners, a quasi-apprentice belonging; among older Weibo audiences, a custodial stance. What differs across these subgroups is less the strength of identification than its form, which is the sense in which digital interaction transforms identity rather than merely increasing it, and which earlier study tended to flatten by treating identification as a single magnitude (Branje, 2022), (Yang et al., 2022). As the subgroup contrasts rest on descriptive comparison rather than on tests of measurement invariance, they are offered as an interpretive proposal awaiting confirmation.

Third, the shaping logic of platforms, algorithms, and user agency is neither one-way nor symmetrical. Algorithms front-load attention but cannot manufacture conviction; platform affordances raise the ceiling of possible engagement, then yield to community climate, which in these data carried the heaviest moderating weight. User agency, often theorized in the abstract, surfaces here as a negotiation between algorithmic offering and self-directed search—a negotiation more visible among trained users, who navigate against the grain of recommendation to reach repertoire they value.

Comparison with prior accounts produces both convergence and friction. The mediation pattern is consistent with existing findings on networked heritage publics and with heritage-experience research in which involvement reaches identification only through an intervening experiential state rather than directly (Yang et al., 2022). The evidence that community climate outweighs platform affordance, by contrast, diverges from the technocentric tilt of earlier digital safeguarding literature, which has tended to treat interface capability as the binding constraint. The age-cohort inversion—younger users converting participation into cognition more efficiently while maintaining lower baseline identification—complicates the generational pessimism pervading policy discourse on traditional music and aligns with developmental studies in which adolescence and young adulthood are marked by active exploration rather than settled commitment (Branje, 2022).

Several alternative explanations cannot be excluded, and they temper the readings above. Selection is the most serious: users who already identify strongly with traditional music may seek out co-creation rather than be changed by it, thereby reproducing the observed ordering with the causal arrow reversed. Algorithmic endogeneity is a second issue, since recommender systems allocate exposure non-randomly, so measured participation depth partly reflects what the platform chose to show. Third, identity and efficacy were both self-reported; the marker-variable check found no evidence of serious inflation, yet shared method variance cannot be eliminated by design (Podsakoff et al., 2024). Fourth, community climate was measured perceptually, so users disposed toward identification may also perceive their communities as warmer. A cross-sectional design cannot adjudicate among these accounts. Longitudinal panels, or experiments in which the depth of participation is manipulated before identification is measured, are the appropriate remedies, and the present results are best read as specifying what such designs should test.

Three risks deserve frank acknowledgment. Cognitive bias accumulates when algorithmic filtering narrows exposure to already-popular repertoire, leaving less circulated forms (ritual music, minority traditions) under-rehearsed in the public imaginary. Cultural misreading intensifies when short-form encoding strips contextual scaffolding, turning a guqin meditation into background ambiance. Homogenization creeps in as creators optimize for platform legibility, smoothing regional and lineage-specific texture into a generic “traditional” aesthetic. None of these is inherent to digital mediation; each is a tractable design problem.

In practice, the findings point toward prioritizing co-creation affordances over polished consumption interfaces, cultivating community moderation that rewards interpretive depth rather than reach, and pairing algorithmic recommendation with curated long-form companions that supply the contextual frames that short clips cannot carry. These are priorities calibrated to associations observed within a single cultural ecosystem, not to general design laws. What the evidence supports for heritage institutions working in comparable settings is a reordering in which audience cultivation deserves attention alongside content digitization rather than after it.

## 6 Conclusion

This study set out to clarify how digital participation in Chinese traditional music translates into cultural identification through a social-cognitive communication mechanism, and to subject that mechanism to empirical scrutiny. The study delivered a four-layer architecture (input, participation, cognitive, and identity) coupled with a staged process model (encoding, decoding, reconstruction, and internalization), formalized through hybrid SEM and attention-weighted neural specifications, with both components estimated and validated on a paired behavioral–survey dataset of 2,346 records spanning music, short-video, and social media platforms.

Three core findings consolidate the contribution. Depth of participation, rather than volume, is what tracks cognitive transmission, with the co-creation and dissemination strata showing indirect effects on identity more than three times those of passive exposure; because the direct path survives, the mediation is partial rather than full. Cultural identity is better read as a hinge than as a terminal outcome, and the chained estimate is consistent with identification feeding back into cognitive processing—a reading that the present design supports as a hypothesis for longitudinal study rather than as an established sequence. Community climate carries the heaviest moderating weight, exceeding platform affordance and content polish, which invites a reordering of the priority assumptions still common in heritage digitization policy.

Theoretically, the study integrates social cognitive theory, cultural identity theory, and contemporary communication frameworks into a single, tractable architecture, providing a measurable mechanism where earlier literature offered only parallel descriptions. It also returns something to psychology in exchange: an account of how mediated participation revises efficacy beliefs, motivational uptake, and the form—not merely the strength—of cultural belonging. Methodologically, the hybrid specification, whose neural component is validated against held-out data rather than asserted, provides a route to treating heritage transmission as both a latent variable and a learning process. Practically, the findings favor investment in community cultivation, co-creation tooling, and curated long-form companions that anchor the contextual meaning short clips cannot carry. These implications are offered as priorities warranted by the present evidence within Chinese-language platform ecologies rather than as universal prescriptions; claims sometimes made for heritage media in the broader language of cultural influence reach well past what a single cross-sectional study can support.

Limitations should be stated plainly, and several bear directly on how much weight the findings can carry. The design is cross-sectional, so the mediation and chained-mediation results describe covariation consistent with the proposed ordering rather than causal sequence; the recursive role attributed to identity is the claim most exposed here, and a simulation study shows that single-wave estimates of mediated effects can depart sharply from the longitudinal parameters they are meant to approximate (Maxwell and Cole, 2007). Cross-lagged panel designs, or experiments that manipulate participation depth before identification is measured, are the appropriate remedies, and the present estimates are best treated as specifying what such studies should target. Sampling was purposive and confined to Chinese-language platforms and predominantly mainland users. Hence, the findings speak to a single cultural ecosystem, and to engaged audiences within it, rather than to a general population. The latent constructs rest on self-report and share a common method; procedural separation and a marker-variable check found no serious distortion, but residual method variance cannot be excluded by design.

Behavioral logs capture observable acts while underrepresenting the affective texture that ethnographic methods would surface, and the mapping from logged acts to participation depth involves coding judgments that, however reliable, remain interpretive. The dynamic expressions in Section 3.3 are not estimated because cross-sectional data identify only their stationary counterparts. Finally, the mechanism treats algorithmic curation as a moderator rather than as an endogenous agent whose own learning dynamics shape what users encounter.

The future study can extend in three directions. Cross-cultural comparison—pairing Chinese traditional music with comparable ICH cases in Japan, Korea, or Southeast Asia—would test the universality of the affordance vs. community ordering. Multimodal data fusion combining audio features, visual cues, network signals, and physiological response would tighten measurement of cognitive reconstruction, and longitudinal designs tracking identity formation across repeated encounters would speak to the developmental dynamics that cross-sectional evidence can only imply (Branje et al., 2021). Intelligent transmission mechanisms, in which recommender systems are co-designed with heritage criteria rather than engagement metrics alone, mark the most consequential frontier; whether such systems can sustain identification without flattening cultural texture is arguably the open question that should guide the next phase of inquiry.

## Data Availability

The original contributions presented in the study are included in the article/supplementary material, further inquiries can be directed to the corresponding author/s.
